# Cyclically Loaded Copper Slag Admixed Reinforced Concrete Beams with Cement Partially Replaced with Fly Ash

**DOI:** 10.3390/ma15093101

**Published:** 2022-04-25

**Authors:** Sumathy Raju, Jagadheeswari Rathinam, Brindha Dharmar, Sasi Rekha, Siva Avudaiappan, Mugahed Amran, Kseniia Iurevna Usanova, Roman Fediuk, Pablo Guindos, Ramkumar Velayutham Ramamoorthy

**Affiliations:** 1 Department of Civil Engineering, Alagappa Chettiar Government College of Engineering and Technology, Karaikudi 630001, Tamil Nadu, India; 2 Department of Civil Engineering, K. Ramakrishnan College of Technology, Trichy 621112, Tamil Nadu, India; sivag616@gmail.com; 3 Department of Civil Engineering, Thiyagarajar College of Engineering, Madurai 625015, Tamil Nadu, India; dbrindha@yahoo.co.in; 4 Department of Civil Engineering, Government College of Engineering, Tirunelveli 627007, Tamil Nadu, India; msasirekha.gce@gmail.com; 5 Departamento de Ingeniería en Obras Civiles, Universidad de Santiago de Chile, Av. Ecuador, Estación Central 3659, Chile; siva.avudaiappan@usach.cl; 6 Centro Nacional de Excelencia para la Industria de la Madera (CENAMAD), Pontificia Universidad Catolica de Chile, Av. Vicuna Mackenna, Santiago de Chile 4860, Chile; pguindos@uc.cl; 7 Department of Civil Engineering, College of Engineering, Prince Sattam Bin Abdulaziz University, Alkharj 16273, Saudi Arabia; 8 Department of Civil Engineering, Faculty of Engineering and IT, Amran University, Amran 9677, Yemen; 9 Peter the Great St. Petersburg Polytechnic University, 195251 St. Petersburg, Russia; plml@mail.ru (K.I.U.); roman44@yandex.ru (R.F.); 10 Polytechnic Institute, Far Eastern Federal University, 690922 Vladivostok, Russia; 11 Indian Plywood Industries Research Institute (IPIRTI), Bengaluru 560022, Karnataka, India; vrramkumar@ipirti.gov.in

**Keywords:** monotonic load, forward cyclic load, stiffness, energy absorption and ductility

## Abstract

Generally, the concrete with higher strength appears to produce brittle failure more easily. However, the use of mineral admixture can help in enhancing the ductility, energy dissipation, and seismic energy in the designed concrete. This paper presents energy absorption capacity, stiffness degradation, and ductility of the copper slag (CS) admixed reinforced concrete with fly ash (FA) beams subjected to forward cyclic load. The forward cyclic load was applied with the help of servo-hydraulic universal testing machines with 250 kN capacity. Twenty-four beams with a size of 100 mm × 150 mm × 1700 mm made with CS replaced for natural sand from 0% to 100% at an increment of 20%, and FA was replaced for cement from 0% to 30% with an increment of 10% were cast. Beams are designed for the grade of M30 concrete. Based on the preliminary investigation results, compressive strength of the concrete greatly increased when adding these two materials in the concrete. Normally, Grade of concrete can change the behaviour of the beam from a brittle manner to be more ductile manner. So, in this work, flexural behaviour of RC beams are studied with varying compressive strength of concrete. Experimental results showed that the RC beam made with 20% FA and 80% CS (FA20CS80) possesses higher ultimate load-carrying capacity than the control concrete beam. It withstands up to 18 cycles of loading with an ultimate deflection of 60 mm. The CS and FA admixed reinforced concrete composite beams have excellent ultimate load carrying capacity, stiffness, energy absorption capacity, and ductility indices compared to the control concrete beam.

## 1. Introduction

Offshore structures are built to withstand both static and cyclic loads caused by ocean waves. Cyclic loading refers to the continuous and repeated load application on material or structural components that causes material degradation and, eventually, fatigue. Materials deteriorate due to fatigue when subjected to cyclic loading. Polymer cement concrete with 5% and 15% cement replacement by ground tire rubber and epoxy exhibited good structural behaviour [1]. The behaviour of unconfined reinforced concrete connections made of sustainable concrete under cyclic loading using various types of sustainable concrete studied. When exposed to cyclic loading, beam-column connections made with iron filings-concrete are significantly damaged, whereas those made with silica fume concrete and fuel ash-concrete are stronger and experience minor cracks [2]. The exterior reinforced beam-column joints made of recycled aggregate concrete mixed with electric arc furnace (EAF) slag aggregate and subjected to horizontal reversed cyclic loading. The seismic performance of joints made with EAF slag concrete was superior to conventional materials [3]. The effects of engineered cementitious composite (ECC) on the behaviour of an RC exterior beam-column joint subjected to reversed cyclic loading was investigated. Compared to the normal concrete specimen at ultimate and failure stages, the ECC joint improved ultimate shear and moment carrying capacities [4]. The behaviour of rubberized concrete in structural applications and to numerically predict the behaviour of rubberized concrete beams and columns. The compressive strain capacity, viscous damping ratio, and kinetic energy of concrete increased as the rubber content increased [5]. Using a higher strength concrete could improve flexural strength, flexural ductility, or both [6]. Because of the high ferrous content of the copper slag (CS), the density of the concrete increased when it was added. Optimum compressive strength was obtained for concrete with 30% cement replacement with fly ash (FA) and 80% fine aggregate replacement with CS. It was 36.8% higher than the strength of the control mix at 28 days [7,8,9,10]. 

The CS in reinforced cement concrete elements increases the compressive, splitting tensile, flexural strength, and energy absorption characteristics significantly [11]. The concrete mixture containing 80% CS exhibited around 48% and 14.7% higher split tensile strength and bond strength than control concrete, respectively [12]. It is possible to produce ultra-high strength concrete of compressive strength more than 150 MPa with 100% untreated CS and 200 MPa with 100% treated CS as a replacement for quartz sand [13]. The use of CS as a cement or natural sand substitute in concrete has lowered both the cost of production and the problem of air pollution [14]. Mortars with larger CS sand contents had lower early strengths at a lower water-cement ratio of 0.48, while mixtures with 20–80% substitution of CS had higher later strengths than the control specimens [15]. Because of the strength qualities of CS and the greater bonding between CS aggregate and the cement paste matrix, using CS as coarse aggregate in concrete improves the mechanical properties of high-strength concrete [16,17].

Similarly, the production of energy is reliant on coal-fired thermal power plants. According to research published by the national thermal power corporation in Noida Uttar Pradesh, India, ash generation is predicted to rise every year due to the current boom in the power sector [18]. A considerable amount of FA is created each year, and if it is not used properly, it can be detrimental to the environment. According to the central electricity authority of India’s report for 2014–2015, India had the greatest level of FA use (63%) in 2009–2010. However, achieving 95–100% usage would necessitate a significant amount of effort [19,20]. 

Reportedly, it was found that the strength of the mix with 40% FA substitution is always lower than that of the control mix [21,22,23,24,25,26,27,28]. However, all FA-replaced combinations develop strength at a faster pace than control mixes, ranging from 10% to 30%. Due to improved pore structure, the strength of the concrete containing roof tiled waste aggregates increased up to 40% of class F FA substitute for cement [29]. The best blend is 30% FA and 70% CS, which is employed in the flexible pavements’ sub-foundation layers. This aids in the preservation of traditional sub-base aggregates and the elimination of problems associated with the disposal of industrial waste such as CS and FA [30,31,32]. It is reported that the compressive strength of blended Portland cement had increased with a reduction on the particle size of FA. It appears that fineness is a more beneficial criterion in increasing the strength gain of FA mortars over chemical composition [21,22,23,24,25,26,27,28]. As the FA fineness of concrete grows, so does its tensile and compressive strengths [33]. Finely powdered pozzolans give mortar an extra strength boost [34,35,36]. Because the sum of alumina, silica, and iron oxide in CS is about 8%, it is a pozzolanic material, exceeding the 70% criterion of calcined natural pozzolans and class N raw [14,37]. According to Sharma [34,35], the inclusion of CS increased the delay in setting time, but did not affect its durability. Moreover, it is reported that the greater the volume proportion of CS as a fine aggregate, the greater the bleeding, due to the heavy specific weight and glass-like surface qualities with uneven grain shape [38,39]. Compressive strength of concrete was significantly increased up to 90 days and capillary porosity decreased due to densification of structures when concrete with 15% CS replaced for cement and 1.5% hydrated lime is used to activate the hydration [37]. Optimum compressive strength and split tensile strength are observed with 35–40% CS and 30–35% CS replaced for fine aggregate with concrete [40]. Compressive (7, 28, and 90 days), flexural, and tensile splitting strength (28 days) recommended less than 40% CS due to insufficiency of cement content in the concrete matrix [34]. Palani et al. [41] has studied on the copper slag by weight replacement of natural sand from 0% to 100% with 10% increment in three different grades (M40, M60, and M80) with constant workability. Strength characteristics such as compressive strength, tensile strength, and flexural strength increase continuously. 

Durability indicators such as water absorption and permeability are commonly decreased continuously. Reportedly, the use of 100% CS showed the best alternative material for sand to attain higher hardened strength properties [42]. It is also reported that water absorption and permeability decreased at 90 days due to a complete hydration of fly ash (FA) [7]. Thus, it is suggested the addition of 100% CS as the alternative materials for natural aggregate with 30% FA. Mucteba et al. [43] has studied on the durability properties of concrete containing class C and class F fly ashes. Three levels of cement replacement with fly ash 10%, 30%, and 50% by weight of cement. Chloride ion permeability resistance increased and sorptivity decreased concrete containing class C and class F fly ash better performance than the control concrete. Chloride ion permeability resistance results are in the range of 131–2982C at 90 days [43]. The presence of a concrete layer with higher strength on the compression fibre of graded concrete (GC) beams is predicted to increase the ductility of the beam. GC can change the behaviour of the beam from a brittle manner to be more ductile which is beneficial for the structure than normal concrete [44].

The use of CS as sand replacement yielded a comparable increase in compressive and flexural strength, and the optimum percentage of CS for mechanical strength and chemical resistance was 60% [45]. The chloride ion penetration in concrete is reduced when 80% of the fine aggregate is replaced with CS and cement is partially replaced with FA. FA is added as a replacement for cement. It reacts with Ca(OH)_2_ in hydrated cement paste to form complex compounds that reduce permeability, prevent corrosion, and improve durability while also improving the economy of the mix [46]. Much research related to CS replaced for either natural sand or replacement for cement. However, few studies are carried out for the behaviour of concrete with waste material or industrial by-products in concrete under cyclic loading. In general, high-strength and high-density concrete is appropriate for reversing loading conditions like high winds and earthquakes. The density of CS mixed concrete is greater than that of conventional concrete. In the preliminary investigation, compressive strength of concrete was greatly increased when adding these two materials in the concrete. Normally, grade of concrete can change the behaviour of the beam from a brittle manner to be more ductile manner. Various approaches have been attempted to increase the ductility of structural elements, either by modifying material properties, modifying the configuration of rebars and detailing and another engineering approach [44]. Based on the previous literature, the objectives of this work are the influence of material properties on flexural behaviour of beams was studied. As a result, a thorough investigation of the behaviour of reinforced cement concrete beams subjected under the monotonic loading and forward cyclic loading conditions were carried out in this work. Peak lateral load, number of cycles, crack pattern, ductility, and stiffness were measured, and results of the tests were compared to the behaviour of conventional concrete.

## 2. Materials and Methods

### 2.1. Materials

Ordinary Portland cement (OPC) 43 grade cement was used in the experimental investigation, which was confirmed with Indian Standard IS8112 Part1: 2013 [47], and physical properties of cement are given in Table 1. A class F fly ash obtained from Thermal Power Plant, Salem (Tamil Nadu), India is used and fly ashes are classified as low calcium fly ash which has satisfied as per the IS3812(part1):2003 [48]. The fine aggregate was first sieved through a 4.75 mm sieve to remove the particles greater than 4.75 mm, then crushed blue granite stones of maximum size 20 mm are used as coarse aggregate as per Indian Standard IS383-1970 [49]. Physical properties of fine aggregate (river sand), copper slag, and coarse aggregate are given in Table 2. Based on the fineness modulus, the CS had higher fineness modulus of 3.6. In this study, CS was used in mix design due to that this mineral admixtures has more dimensional stability, which will improve impact resistance [31,50]. Similarly, it is reported that the integration of CS and FA as mineral admixtures can help at meeting the required the strength and durability criteria in the mass of the designed green concrete [51]. It could be inferred from the results, the CS was coarser than the fine aggregate. Locally available portable water was used for mixing and curing. M30 grade concrete was designed to prepare all the beams, and concrete mix design was prepared as per Indian Standard IS10262-2009 [52]. There are twenty-four mix proportions studied by varying the proportion of CS and FA for natural sand and cement, respectively.

**Figure 1:**
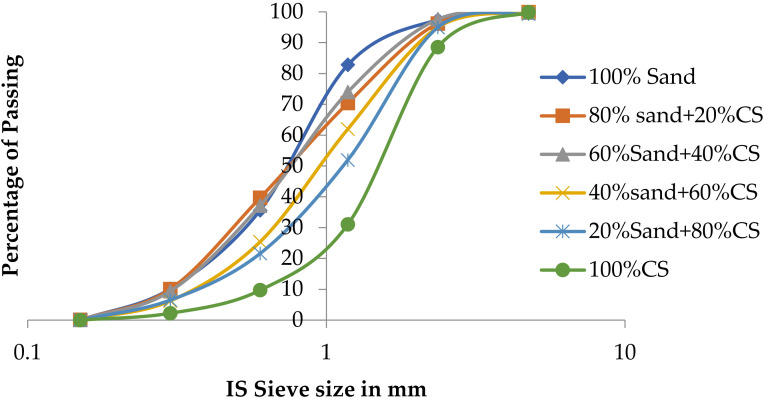
Gradation curve.

The gradation of an aggregate affects both the fresh and hardened concrete properties. The well-graded aggregate sample contained minimum voids, and, also, minimum paste required to fill the void in the aggregate. The gradation curve of fine aggregate was compared with the curves obtained from fine aggregate replaced with CS from 0% to 100% with 20% increment. Table 3 presents the grading of different combinations of fine aggregate and CS. Figure 1 presents the gradation curves of fine aggregate.

**Figure 2:**
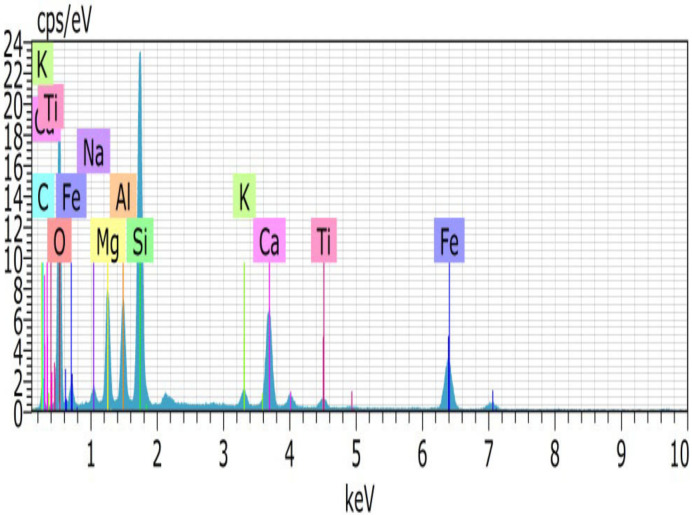
EDAX images of FA.

**Figure 3:**
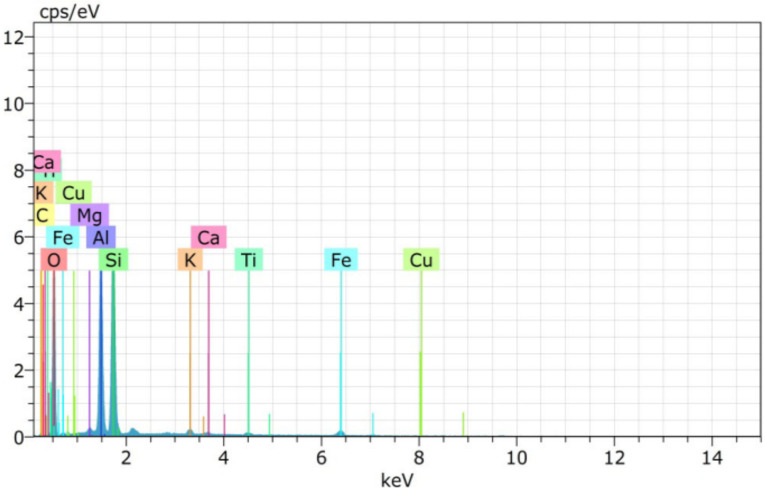
EDAX images of CS.

From the particle size distribution, the fineness modulus for fine aggregates are 2.79, 2.8, 2.83, 3.10, 3.20, and 3.52, respectively, for 0%, 20%, 40%, 60%, 80%, and 100% CS replacement for fine aggregate. Introduction of CS shifts the gradation curve towards right because the fineness modulus values are increased. Microstructure study was conducted by EDAX (energy dispersive analysis of X-ray) for FA and CS. EDAX shows that the major components are Ca, Si, Fe, Mg, and Al and the minor components are Na, Ti, K, and Pd. EDAX images of CS and FA are shown in the Figure 2 and Figure 3. Chemical components of FA and CS are given in Table 4. Also, the mix composition of M30 grade of concrete is given in Table 5.

### 2.2. Methods

#### 2.2.1. Preliminary Investigation

Mix design is prepared for M30 grade of concrete according to IS: 10262-2009. Twenty-four mixes are prepared in the laboratory where cement is partially replaced by FA from 0, 10, 20, and 30%, (by mass), respectively, and fine aggregate is replaced by CS from 0, 20, 40, 60, 80, and 100%. Compressive strength test has been conducted on 432 cubes of size 150 mm × 150 mm × 150 mm at 3, 7, 14, 28, 56, and 90 days curing period using a compression testing machine (LABTEST, Kirti Nagar, New Delhi, India) with 2000 kN capacity (see Table 6). Quality of concrete was verified by both destructive testing (DT) and non-destructive testing (NDT). Based on 90 days compressive strength, concrete mixtures with 30% of FA and 100% of CS have contributed higher strength than the control mix for all the time. Optimum strength is reached, when concrete with cement is replaced by 30% of FA and 80% of CS for fine aggregate. It is 36.83% better than the strength of control mix and also this mix proportion is suitable for concrete structures [7]. Durability properties such as saturated water absorption, porosity, coefficient of water absorption, sorptivity, rapid chloride ion penetration test, and alkalinity (pH) test were also conducted for twenty-four mix proportions. Based on the RCPT test shows that, according to ASTM [C1202-12], the chloride ion penetration is very low and this mixture is suitable for seashore areas [53]. Five reinforced concrete (RC) beams of size 150 mm × 250 mm × 3200 mm were cast based on the optimum mix proportion and flexural behaviour of RC beams was monitored by a four-point bending test with a load carrying capacity of RC beams were increased by 80 to 100% CS replaced for natural sand, and 30% FA replaced for cement [54].

#### 2.2.2. Beam Design and Preparation

**Figure 4:**
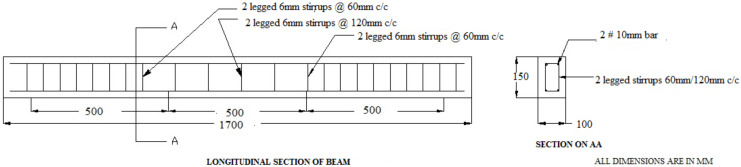
Reinforcement details and specimen casting.

Each beam had a cross section of 100 mm × 150 mm and 1700 mm long. High yield strength deformed (HYSD) Fe415 steel bars are used as reinforcements The main reinforcements were two 10 mm diameter deformed bars for both tension and hanger bar. The 6 mm diameter bars are used as stirrups at 60 mm spacing at shear span and 120 mm spacing (double spacing) in pure bending zone. All the specimens were designed as under-reinforced beams as per the Indian Standard IS456-2000 [55]. All the beams have an effective span of 1500 mm, shear span to depth ratio of 4.0 and a clear concrete cover of 25 mm. Details of reinforcement represented in the Figure 4. Totally, twenty-five reinforced concrete beams were cast in this investigation. The reinforcement cage was placed in cast ion mould and the proper cover was given to the reinforcing bars to maintain 25 mm cover thickness. Then, the concrete was mixed with help of titling drum laboratory mixer machine without hopper with 3hp motor. The mixer machine was purchased from M/s Asian Tools and Instruments, New Delhi, India. The concrete was placed in two to three layers, and each layer was compacted by using a vibrating table. The specimens are demoulded after 24 h followed by curing process in the curing pond. 

#### 2.2.3. Loading Setup 

**Figure 5:**
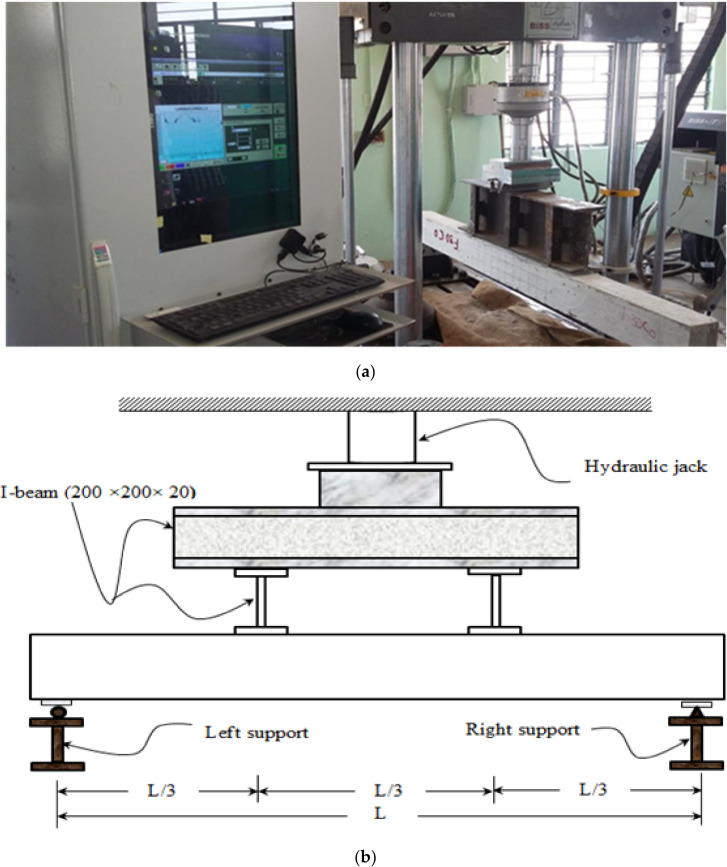
Typical setup of servo-hydraulic universal testing machines. (**a**) Experimental setup. (**b**) A graphic representation of the calculated beam loading schema.

The beam spanning 1500 mm was subjected to four-point bending flexural testing. The load was applied with help of servo-hydraulic universal testing machines fitted with a 250 kN capacity load cell and 300 kN actuator. The servo-hydraulic UTM was purchased from M/s AIMIL Ltd., New Delhi, India. Both monotonic and cyclic loadings are applied to the control reinforced concrete beam. Flexural behaviours of the remaining beams are tested by cyclic load only and they are compared with the flexural behaviour of the control reinforced concrete beam. Spreader beam was placed over a concrete beam at the distance of l/3 points from each support as shown in Figure 5a,b. The test setup is represented in the Figure 5. 

#### 2.2.4. Testing Procedure

Forward cyclic load was applied on the specimen by using servo-hydraulic UTM 250 kN capacity with MTL32-2020 software (BISS, Bangalore, India). The beam was gradually loaded by increasing the load level in each cycle. The load was applied from 0 to ultimate level at an increment of 2 kN (i.e., 2 kN, 4 kN, etc., up to the failure of the beams). The deflection of the beam was measured for every load increment. The beam was loaded up to the first increment, then unloaded until the force equal to zero and reloaded to the next increment of load and vice versa. This procedure was followed up to failure of the specimen and this pattern of loading will be continued for all twenty-four beams up to failure. The first crack was marked on the specimen and simultaneously, the no of cycles was noted down for the corresponding first crack. Yield load, ultimate load carrying capacity, energy absorption, ductility index, and stiffness of concrete beams and modes of failure are considered in this study.

## 3. Results and Discussions

### 3.1. Monotonic and Forwarded Cyclic Load

In earthquake resistant design, a prime consideration is about the ability of the structure to deform in a ductile manner, when subjected to several cycles of lateral loading well into the inelastic range. Monotonic load and forward cyclic load are applied to the control concrete RC beam. Remaining beams are loaded by forwarded cyclic load and compared with control concrete beam subjected to cyclic loading condition.

**Figure 6:**
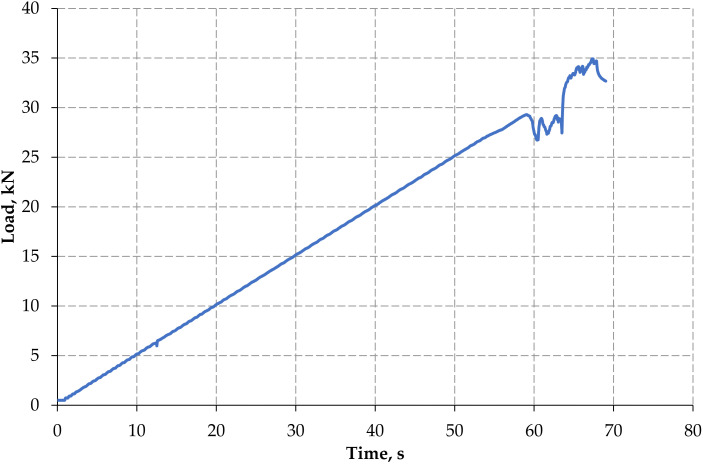
Load history—monotonic load.

**Figure 7:**
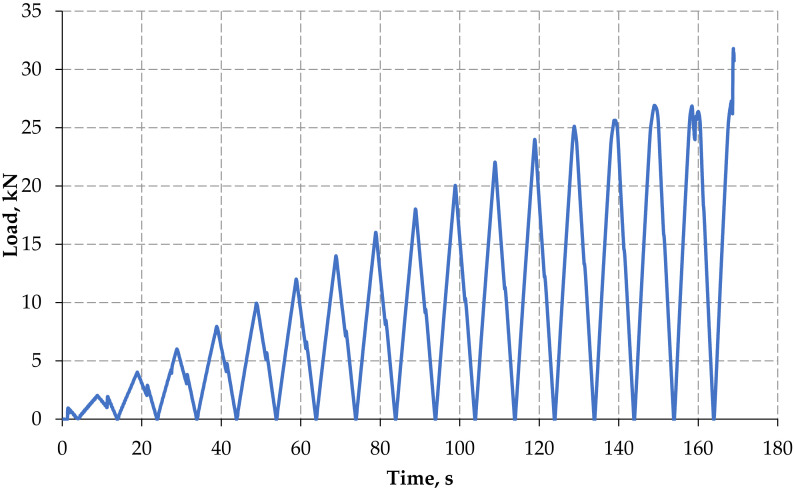
Load history—forward cyclic load.

Load history for monotonic and forwarded cyclic loads is represented in Figure 6 and Figure 7. In control concrete beam for monotonic loading condition, the first crack was formed at the load of 15.17 kN, and it withstands up to 35 kN, whereas in cyclic loading condition the first crack was developed at 9.94 kN, and survive up to 31.78 kN only.

### 3.2. Load–Deflection Behaviour

**Figure 8:**
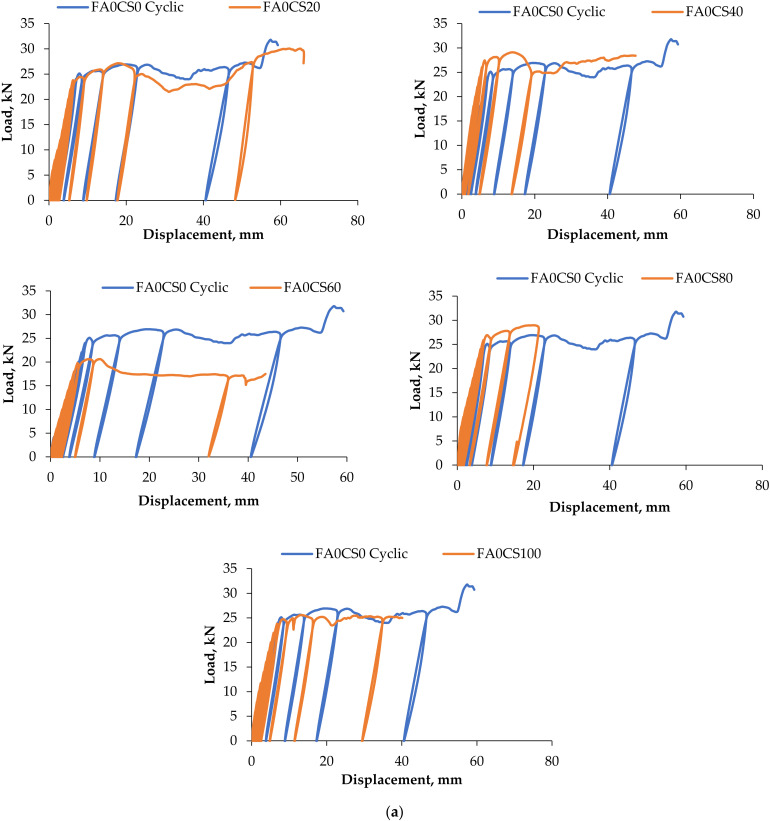

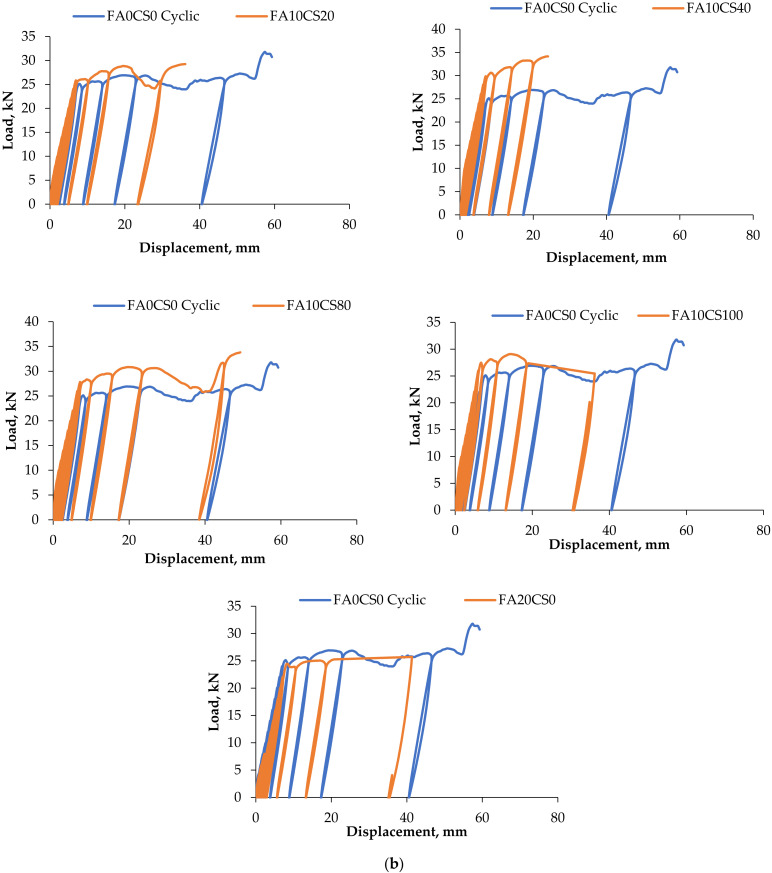

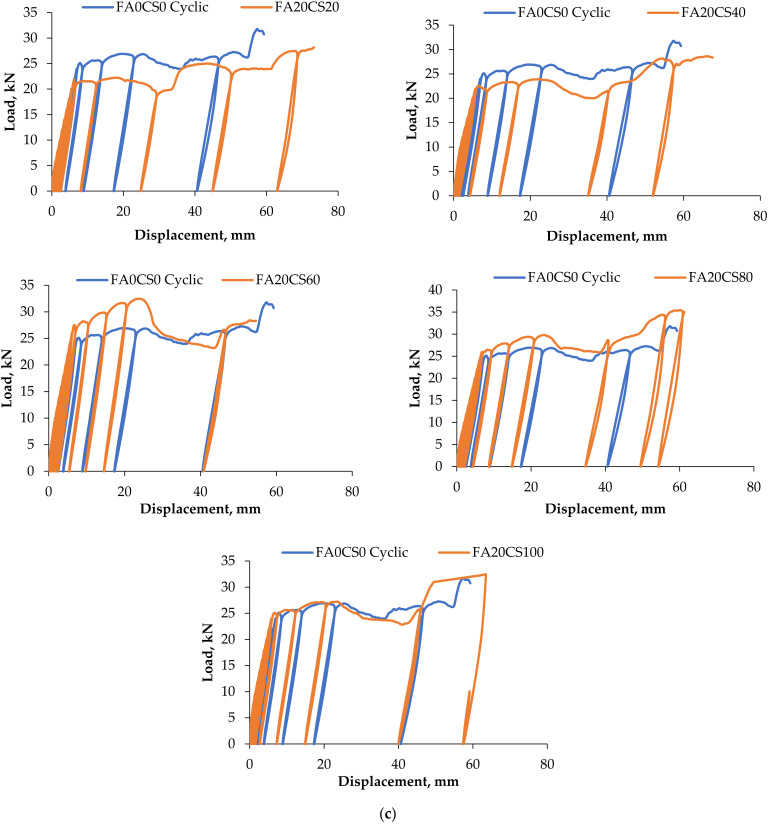

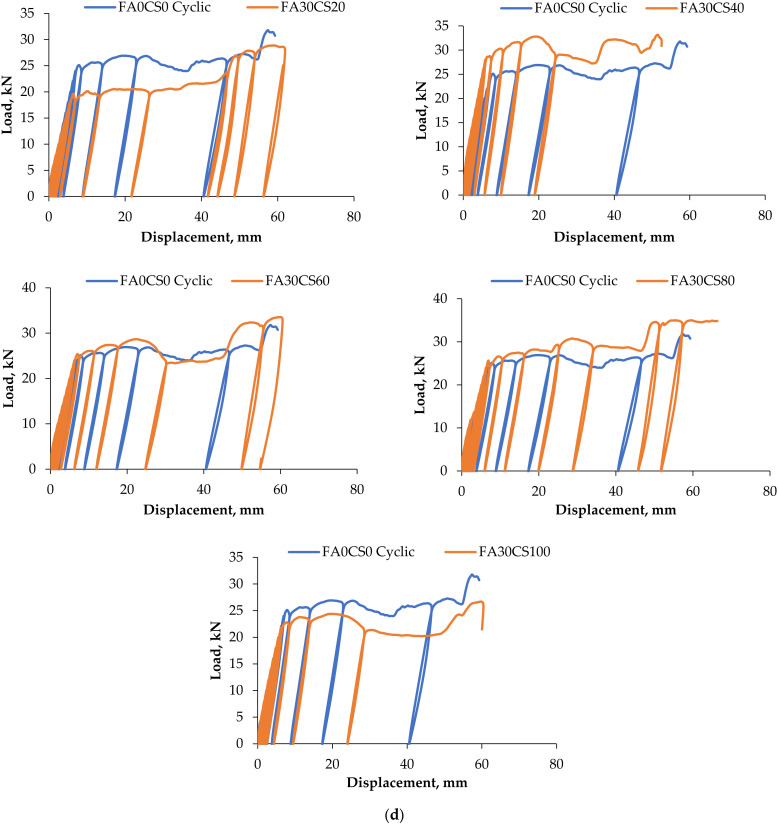
Load and deflection curves: (**a**) Batch I: The addition of 0% FA and 20–100 CS, (**b**) Batch II: the addition of 10% FA and 20-100 CS, (**c**) Batch III: the addition of 30% FA and 20-100 CS, (**d**) Batch IV: the addition of 3% FA and 20-100 CS.

The load–displacement curve at mid span section is an important factor for the evaluation of mechanical behaviour of simply supported reinforced concrete beam. So that the mid-span deflection of the reinforced concrete composite (RCC) beam with cyclic loading was compared with control concrete beam and represented in Figure 8. This figure depicts the load deflection behaviour of twenty-four beams compared with the control beam. On repeated cycles of loading and unloading, the slope of the curves was successively decreased; it means stiffness of the beam is decreased with a number of cycles. All the beams are subjected to cyclic loading up to failure with a gradual increment from 0 to 2 kN. The loading and reloading curves does not match with the initial curves. This indicates that the occurrence of stiffness degradation. Load at first crack, ultimate load, no. of cracks, no. of cycles carried at first crack, and ultimate load are listed in Table 7.

From the load–deflection curve (Figure 8a), it can be understood that 0–100% CS and 0% FA admixed RCC beams are stiffer than the control beam. In control concrete beam, the first crack was developed at the fifth cycle and the first crack was initiated very early compared to concrete with FA and CS, due to the limited tensile strength of the concrete. The yield and ultimate load carrying capacities of concrete with CS and FA admixed beams are higher than the control of concrete beams. The first yield deflection was assumed as the bilinear behaviour of the beam. Even though the deflection of CS and FA admixed beams was slightly higher than the control concrete beams, the load carrying capacity of admixed beams was still greater than the control beam. All of the beams were deflected the most at mid-span. The lateral beam deflection at mid-span became less substantial than that at the top once cracking appeared. However, crushing was the sole cause of total failure throughout testing. There was no evidence of local buckling or overall instability.

From the load–deflection curve (Figure 8b), it can be seen that 0–100% CS and 20% FA admixed RCC beams are stiffer than the control beam. At 45% of the ultimate load, Figure 8b showed a similar elastic trend. After the load was increased, there were minor alterations in the plasticity behaviour. It is detected that the first crack was developed at the fifth cycle and the first crack was initiated very early compared to concrete with 10% FA and 0–100% CS, due to the limited tensile strength of the concrete. As a result, this system can be employed in flooring applications to form slab systems. The higher slender beam, on the other hand, buckled violently, whereas the less slender beams buckled due to crushing near the top and bottom margins. Even though the deflection of CS and FA admixed beams was slightly higher than the control concrete beams, the load carrying capacity of admixed beams was still greater than the control beam. (b) Batch II: The addition of 10% FA and 20–100 CS. From the load–deflection curve (Figure 8c), it can be found that 0–100% CS and 20% FA admixed RCC beams are stiffer than the control beam. In the control concrete beam, in the first testing stage, until the first crack emerged at roughly 50% of the failure load, and in the mid-span lateral deflection of 15% of the ultimate deflection, Figure 8c revealed a linear elastic behaviour. At this point, the first flexural crack was discovered. Each flexural crack in the load–deflection plot developed in response to a different load drop. Beyond the second flexural crack load, the deflection mode rapidly increased for a given load increase. Even though the deflection of CS and FA admixed beams was slightly higher than the control concrete beams, the load carrying capacity of admixed beams was still superior compared to the control beam. At the ultimate deflection mode, the panels were loaded until ultimate failure. (c) Batch III: The addition of 30% FA and 20–100 CS. From the load–deflection curve (Figure 8d), it can be observed that CS and FA admixed RCC beams are stiffer than the control beam. All of the beams’ loads versus mid-span deflection curves are shown in Figure 8d (30% FA and 0–100% CS). As the load grew, the deflection increased linearly up to a particular load, called the yield load. The mid-span deflection fluctuated non-linearly before reaching its maximum magnitude. As the load decreased past the ultimate load point, the deflection began to grow noticeably. Until the load of the first crack, the panels behaved in a linear elastic way during the early stages of testing. Each flexural crack’s progression in the load–deflection plot was linked to a noticeable decline in the load–deflection curve. The beams were loaded until they reached the ultimate failure load. Albeit that the deflection of 20% CS and 30% FA admixed beams was slightly higher than the control concrete beams. (d) Batch IV: the addition of 3% FA and 20–100% CS.

The specimens with FA 20% to 30% with CS 80% have achieved 35.4 kN and 34.98 kN and their corresponding deflections are 60 mm and 55.24 mm, respectively. This shows that ductile behaviour was high for RCC beams incorporating CS and FA admixed beams. It was observed that the maximum failure load has been obtained for (FA20CS80) beam, and it was 35.40 kN, whereas for control concrete beam, it was 31 kN only.

From these figures, it can be seen that, in the initial stage of loading the specimen was in elastic range. Hence the area under the load–displacement curve was very small. With the increase in load, the specimen transferred from elastic range to elastic-plastic range then automatically formation of crack will occur. The increase in damage also observed with the decrease in stiffness of the specimen. At last, the area under the load–displacement curve increased successively. This is also an indication for dissipation of energy during the loading and unloading process. CS replacement does not affect the load carrying capacity [56].

### 3.3. Stiffness Degradation

**Figure 9:**
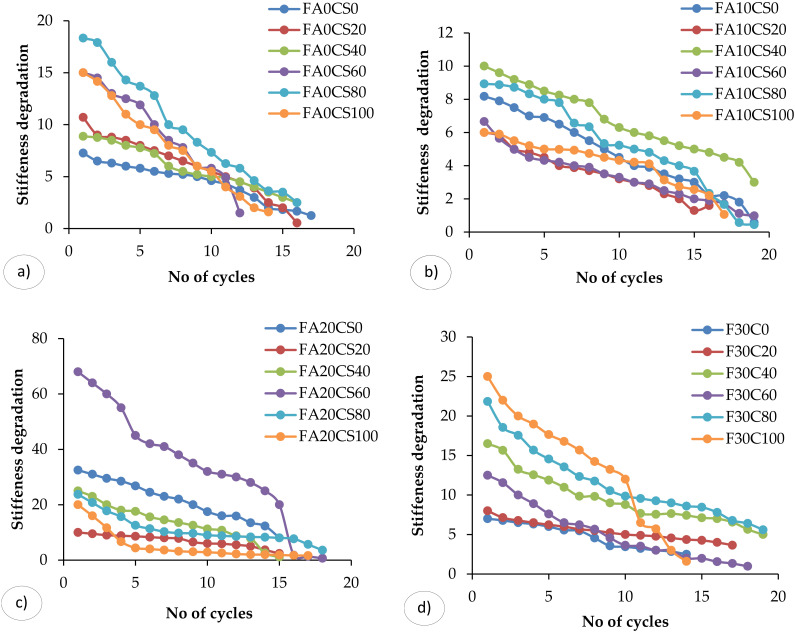
Stiffness degradation versus no. of cycles: (**a**) 0%FA and 0–100%CS, (**b**) 10%FA and 0–100%CS, (**c**) 20%FA and 0–100%CS, (**d**) 30%FA and 0–100%CS.

Stiffness is the main variable controlling safety against instability. Stiffness is defined as the load required causing unit deflection of the RC beam. Generally, the increase in displacement degrades the Stiffness. Figure 9 depicts the stiffness versus cycles to failure trends for all stacking sequences tested. All of the RC members will leads to stiffness degradation when it is subjected to no of cycles of loading and unloading. The stiffness degradation occurs due to the formation of cracks, loss of bond, and interaction with high axial or shear stresses. However, the amount of stiffness degradation mainly depends on characteristics of RC member and load increment. The ultimate stiffness, initial stiffness and percentage reduction in stiffness are calculated and represented in the Figure 9. 

The stiffness of the structural members gradually decreases with increasing cycles of loading. The beam FA30CS20 (see Figure 9d) has 34.8% more stiffness compared to the control concrete beam, and, also, the percentage reduction is less. The FA20CS60 (Figure 9c) was demonstrated the higher stiffness degradation compared to the other beams. The stiffness deterioration decreased rapidly during the initial cycles in all four stacking sequence scenarios. Fatigue cracks of composite samples appeared to be extremely sensitive at the imposed load level. FA10CS0-100 (Figure 9b) had the lowest rates of degradation, while FA0CS0 (Figure 9a) demonstrated the lowest rate of depreciation. A minute and rapid stiffness degradation curve was detected for all four components, which explored the damage progression. A small and abrupt stiffness degradation curve was discovered, which analysed damage development.

### 3.4. Energy Absorption Capacity

**Figure 10:**
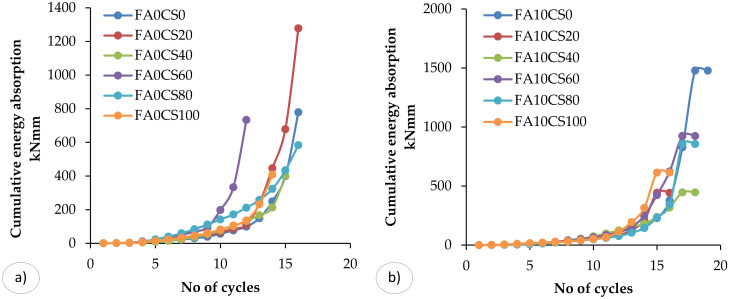

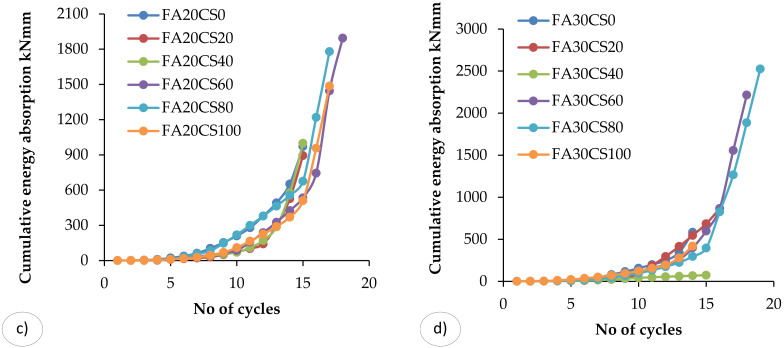
Cumulative energy absorption versus no. of cycles: (**a**) 0%FA and 0–100%CS, (**b**) 10%FA and 0–100%CS, (**c**) 20%FA and 0–100%CS, (**d**) 30%FA and 0–100%CS.

One of the most important parameter of the structure under seismic loading is energy dissipation capacity. It is defined as the ability of the structure to dissipate the energy. Generally, the energy absorption capacity of the beam members can be approximated as the area is enclosed by the hysteresis loop (load–deflection curve). When the beam is subjected to cyclic loading such as those experienced during heavy wind or earthquake, some energy is absorbed in each cycle (see Figure 10). It is equal to the work in straining or deforming the structure to the limit of deflection. The energy absorption capacities during various load cycles are calculated from the load versus deflection curve. When the beam undergoes deflection under loading, it tends to absorb some energy. It denotes the total amount of strain energy released during the failure of the specimen. The trend in stiffness deterioration was used to evaluate crack progression under cyclic loading. In composite materials, fatigue damage always reduces stiffness rather than composite strength. The cumulative energy absorption capacity of the beam specimen is obtained by adding the energy absorption capacity of the specimen during each cycle considered and it is showed in Figure 10. The beam FA30CS40 (Figure 10d) has 90.66% lower energy absorption capacity than the control beam, whereas the beam FA30CS80 (Figure 10b) has 69.2% higher energy absorption capacity than the control beam. 

Moreover. the beams FA0CS0-100 (see Figure 10a) has showed the most rigidity than the control concrete beam, and, also, has a lower percentage reduction. Additionally, the beams FA10CS0-100 (Figure 10d) displayed the greatest stiffness degradation. In all four stacking sequence scenarios, stiffness degradation declined fast during the initial cycles. At the applied stress level, fatigue cracks in composite samples proved to be particularly sensitive. The beams FA10CS0-100 (Figure 10b) had the moderate degradation rates, while beams FA20CS0-100 (Figure 10c) had the second higher rate of deterioration. In brief, the addition of FA content has a significant impact in improving the stiffness of the designed beams.

### 3.5. Ductility Factor

**Figure 11:**
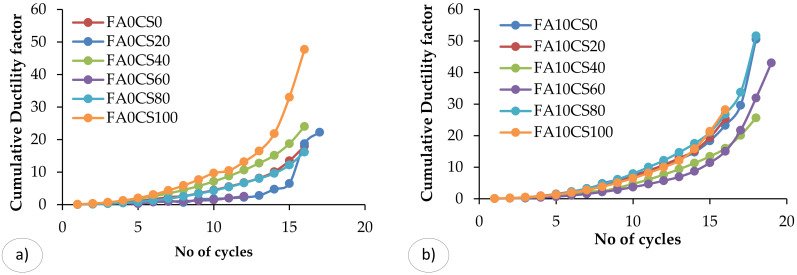

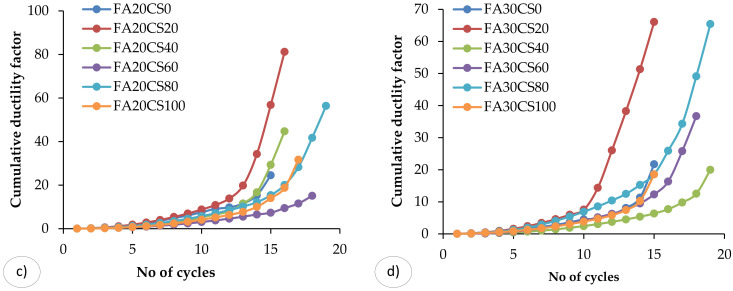
Cumulative ductility factor versus no. of cycles: (**a**) 0%FA and 0–100%CS, (**b**) 10%FA and 0–100%CS, (**c**) 20%FA and 0–100%CS, (**d**) 30%FA and 0–100%CS.

Ductility is the ability of the structure which is used to compensate the brittle failure. Ductility of a structure or its members is also defined as the capacity to undergo large inelastic deformation (beyond the initial yield deformation), without significant loss of strength or stiffness. If a structure is ductile, it can be expected to adapt to unexpected overloads, load reversals, impact, and structural moments, due to the foundation settlement and volume of changes. Secondly, if the structure is ductile, its occupants will have sufficient warning of the impending failure, thus reducing the probability of loss of life in the event of a collapse. In this work, ductility factor (see Figure 11) is defined as the ratio of maximum deflection obtained in each cycle to the yield deflection. The yield deflection was determined from the assumed bilinear load deflection curve [57,58,59,60]. The ductility factor µ, a measure of ductility of a structure, is defined as the ratio of ∆u and ∆y, where ∆u and ∆y are the respective lateral deflections at the end of the post elastic range and when the yield is first reached. Ductility factor is calculated from the Equation (1).

µ = ∆u/∆y (1)

Ductility index is used to determine the ductile nature of the beam. It can be seen that FA20CS20 (Figure 11c) is more ductile in nature compared to the control concrete beam and FA30CS40 (Figure 11d). The beam FA20CS20 has 77.85% higher ductility factor than the control beam whereas the beam FA0CS60 (Figure 11a) has 37.22% lower ductility factor than the control beam and higher than FA10CS40 (Figure 11b). Low-density concrete is also undesirable, due to its relatively poor performance under reversed cyclic loading.

### 3.6. Failure Pattern

**Figure 12:**
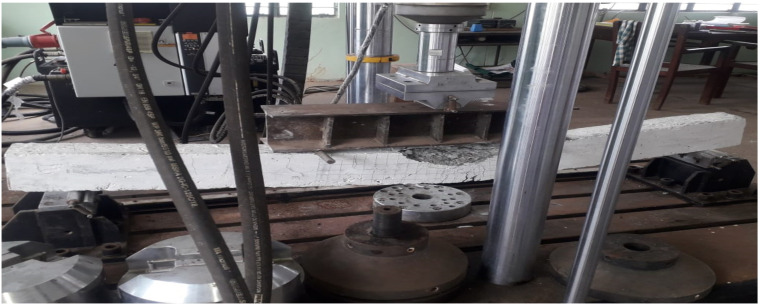
Failure pattern of RC beams.

Failure pattern of RC beams were depicted in the Figure 12. At about 31% of the ultimate load, well flexural cracks have appeared at the bottom of the specimen. With further increase in the load, regularly spaced vertical cracks were observed and they extend from the bottom of the specimen where the tensile stress exceeds the tensile strength of the concrete towards the top of the specimen. It was observed that all the cracks are formed within the flexural region. Hence, all the beams were failed under flexure mode. Mode of failure was under reinforced section. 

This type of admixed beams may be recommended for the structures located in seismic prone areas. At ultimate load condition, the beams fail with the crushing of concrete at the top compression region. However, none of the beams has a shear failure. It indicates that the shear reinforcement provided was sufficient to carry the shear.

## 4. Comparison of Experimental Results with Previous Literatures

Preliminary investigation results are supported by the work of other researchers who studied the influence of CS as fine aggregate on the strength of mortars, concrete specimens, durability studies, and flexural behaviour of RC beams. Compressive strength of concrete significantly increased up to 90 days, and capillary porosity decreased due to densification of structures when concrete with 15% CS replaced for cement and 1.5% hydrated lime is used to activate the hydration [37]. Optimum compressive strength and split tensile strength were observed with 35–40% CS, and 30–35% CS replaced for fine aggregate with concrete [40]. Compressive (7, 28, and 90 days), flexural, and tensile splitting strength (28 days) recommended less than 40% CS due to insufficiency of cement content in the concrete matrix [34]. Palani et al. (2014) has studied on the copper slag by weight replacement of natural sand from 0% to 100% with 10% increment in three different grades (M40, M60, and M80) with constant workability [41]. Strength characteristics such as compressive strength, tensile strength, and flexural strength increase continuously. Durability indicators such as water absorption and permeability decrease continuously. Suggested that up to 100% CS is the best alternative material for sand. Compressive strength values are increased when increasing the curing period of concrete. Higher compressive strength was observed at 90 days curing period for 100% CS replaced for fine aggregate, and 30% FA replaced for cement. Optimum compressive strength was reached for concrete with 30% FA replaced for cement and 80% CS replaced for fine aggregate. Optimum tensile strength has been achieved concrete with, 30% FA replaced for cement, and 40% CS replaced for fine aggregate. Mucteba [43] has studied on the durability properties of concrete containing class C and class F fly ashes. Three levels of cement replacement with fly ash 10%, 30%, and 50% by weight of cement. Chloride ion permeability resistance increased and sorptivity decreased concrete containing class C and class F fly ash better performance than the control concrete. Chloride ion permeability resistance results are in the range of 131–2982C at 90 days [43]. The water absorption and permeability decreases at 90 days due to complete hydration of fly ash (FA), and suggested that 100% CS is the alternative materials for natural aggregate with 30% FA. Rapid chloride ion penetration in the ranges from 101.7 to 801.9C at 90 days [53]. The presence of a concrete layer with higher strength on the compression fibre of graded concrete (GC) beams is predicted to increase the ductility of the beam. GC can change the behaviour of the beam from a brittle manner to be more ductile which is beneficial for the structure than normal concrete [44]. The RC beam FA30CS80 has displayed most ductile behaviour, with a ductility index 4.93 due to high strength concrete give higher ductility. From this list, it is observed that the FA0CS0 beam exhibits less ductility index (3.067) [8].

## 5. Conclusions

In reinforced concrete design, only the strength of the concrete with compression was taken into consideration. Based on the previous research, concrete with CS replaced for fine aggregate gives higher strength than the control concrete. In this work, detailed study was carried out on the RC beams made with different mix proportions subjected to forward cyclic loading. This paper presents energy absorption capacity, stiffness degradation, and ductility of the copper slag (CS) admixed reinforced concrete beams with fly ash (FA) subjected to forward cyclic load. The forward cyclic load was applied with help of servo-hydraulic universal testing machines with 250 kN capacity. Based on the experimental investigation the following conclusions were made.

- During the monotonic loading condition, the control concrete RC beam withstands the ultimate load of 35kN. The first crack was observed at the load of 15.17 kN. It was found that when the specimen loaded under cyclic loading condition, the control concrete beam withstands the ultimate load of 31.78 kN, and the first crack was observed at the load point of 9.94 kN.
- It was found that the specimens with 20% FA and 80% CS (FA20CS80) possesses higher ultimate load carrying capacity compared to the control concrete beam. It withstands up to 18 cycles of loading with ultimate deflection which was 60 mm.
- The stiffness of the structural members gradually decreases with increasing cycles of loading. RCC beam FA30CS20 has 34.78% more stiffness compared to the control concrete beam, and, also, the percentage reduction is less.
- The beam FA20CS20 has 77.85% higher ductility factor than the control beam, whereas the beam FA0CS60 has 37.22% lower ductility factor than the control beam. The beam FA30CS40 has 90.66% lower energy absorption capacity than the control beam, whereas the beam FA30CS80 has 69.2% higher energy absorption capacity than the control beam.
- The CS and FA admixed RCC beams have shown excellent ultimate load carrying capacity, stiffness, energy absorption capacity, and ductility factor compared to the control concrete beam. Because, the density and the stiffness of concrete are increased, due to irregular surfaces of CS filled with hydration products.
- At ultimate load condition, the beams fail with the crushing of concrete at the top compression region. However, none of the beams has a shear failure. It indicates that the shear reinforcement provided is sufficient to carry the shear.
- It is also proposed that when determining the CS replacement amount, the required concrete compressive strength be taken into account.

Based on the major findings of this study, it is recommended to perform a wide-range of theoretical analysis and numerical modelling for this designed system of RC beams.

## Figures and Tables

**Table 1:** Physical properties of cement.

Physical Properties	Cement
Specific gravity	3.15
Normal consistency (%)	34%
Initial setting time (minutes)	90
Final setting time (minutes)	420

**Table 2:** Physical properties of fine aggregate (river sand), copper slag, and coarse aggregate.

Specification	Fine Aggregate	Copper Slag	Coarse Aggregate
Specific gravity	2.51	3.52	2.75
Fineness modulus	2.79	3.6	7.6
Bulk Density kg/m^3^	1420	1750	1380
Void ratio	0.77	0.8	0.95
Water absorption %	1.08	0.13	0.45

**Table 3:** Grading of different combinations of fine aggregate and CS.

IS Sieve Size	Cumulative Percentage Passing, %					
	100% Sand	80% Sand and 20% CS	60% Sand and 40% CS	40% Sand and 60% CS	20% Sand and 80% CS	100% CS
4.75	99.9	99.92	99.76	99.66	99.29	99.85
2.36	97.4	96.21	97.60	94.96	94.94	88.55
1.18	82.8	70.41	73.98	61.99	51.95	31.05
0.6	35.6	39.70	37.15	25.42	21.60	9.7
0.3	10.1	10.05	9.38	6.22	6.47	2.2
0.15	0.03	0.06	0.05	0.03	0.03	0.011

**Table 4:** Chemical components of fly ash and copper slag.

Chemical Components	Fly Ash	CS
O	50.65	45.96
Si	18.67	12.87
Fe	3.07	9.73
Ca	10.2	8.79
C	6.49	8.55
Mg	0.22	5.73
Al	19.6	4.59
Na	-	1.31
Ti	0.47	1.27
K	0.83	1.19

**Table 5:** Mix composition of M310 grade of concrete.

Mix Identification	Cement kg/m^3^	Fly Ash kg/m^3^	Fine Aggregate kg/m^3^	Copper Slag kg/m^3^	Coarse Aggregate kg/m^3^	Water kg/m^3^
FA0CS0	380	-	596	-	1293	152
FA0CS20	380	0	520	183	1293	152
FA0CS40	380	0	390	366	1293	152
FA0CS60	380	0	260	549	1293	152
FA0CS80	380	0	131	738	1293	152
FA0CS100	380	0	0	922	1293	152
FA10CS0	342	38	596	0	1293	152
FA10CS20	342	38	520	183	1293	152
FA10CS40	342	38	390	366	1293	152
FA10CS60	342	38	260	549	1293	152
FA10CS80	342	38	131	738	1293	152
FA10CS100	342	38	0	922	1293	152
FA20CS0	304	76	596	0	1293	152
FA20CS20	304	76	520	183	1293	152
FA20CS40	304	76	390	366	1293	152
FA20CS60	304	76	260	549	1293	152
FA20CS80	304	76	131	738	1293	152
FA20CS100	304	76	0	922	1293	152
FA30CS0	266	114	596	0	1293	152
FA30CS20	266	114	520	183	1293	152
FA30CS40	266	114	390	366	1293	152
FA30CS60	266	114	260	549	1293	152
FA30CS80	266	114	131	738	1293	152
FA30CS100	266	114	0	922	1293	152

**Table 6:** Compressive strength of the designed concrete.

Mix Identification	Average Compressive Strength, MPa						Rate of Strength Development
	(σ_c_)^3^	(σ_c_)^7^	(σ_c_)^14^	(σ_c_)^28^	(σ_c_)^56^	(σ_c_)^90^	
FA0CS0	30.22	38.22	39.77	43.25	47.7	50.07	0
FA0CS20	30.66	33.41	35.25	38.22	47.11	49.78	↓ 0.6
FA0CS40	31.55	36.7	39.55	44.74	48	53.33	6.5
FA0CS60	23.85	27.11	36.44	38.81	41.04	44.14	↓ 11.8
FA0CS80	24.15	27.55	35.11	39.7	42.59	45.92	↓ 8.3
FA0CS100	24.15	25.77	29.04	33.77	38.52	44.88	↓ 10.4
FA10CS0	26.66	29.77	35.73	37.33	46.52	48.59	↓ 7.7
FA10CS20	27.55	37.34	42.22	44.74	46.11	48.9	↓ 2.7
FA10CS40	30.66	44.59	45.77	48.9	50.52	57.03	11.3
FA10CS60	26.22	40.59	42.66	44.45	47.8	55.91	4.8
FA10CS80	24.44	32	39.55	41.9	48	51.56	16.9
FA10CS100	23.55	37.77	40.25	45.32	49.03	51.85	11.0
FA20CS0	17.77	28.68	33.77	34.67	39.11	46.22	↓ 13.0
FA20CS20	18.66	35.11	36.88	37.93	46.23	48.7	1.3
FA20CS40	20.88	36	37.77	44.7	52.01	55.73	3.0
FA20CS60	20.88	36.91	37.77	43	48.01	52.45	0.3
FA20CS80	16.44	34.67	38.22	51.23	53.92	58.51	17.8
FA20CS100	16	33.18	36	45.48	47.27	55.6	12.7
FA30CS0	16	29.33	35.85	36.56	40.44	43.55	↓ 7.7
FA30CS20	16.88	31.55	34.81	38.22	47.85	50.7	↓ 2.7
FA30CS40	24.88	27.55	33.33	43.26	50.24	51.57	11.3
FA30CS60	25.33	26.52	36.44	40.44	41.77	50.2	4.8
FA30CS80	25.77	40.45	40.88	46.5	55	58.96	16.9
FA30CS100	21.77	39.55	40	47.7	55.11	56.44	11.0

Annotations: ↓ − rate of decreasing on the developed strength, ↑ − rate of increasing on the developed strength.

**Table 7:** Experimental results of cyclic loadings and their deflection.

Mix Identification	Load at First Crack (kN)	Deflection at First Crack (mm)	No. of Cycles at First Crack	Ultimate Load kN	Ultimate Deflection in mm	No. of Cycles at Ultimate Load	No. of Cracks
FA0CS0	9.94	2.49	5	31.78	57.35	16	11
FA0CS20	14.02	3.48	7	28.44	62.29	14	12
FA0CS40	14.00	2.32	7	28.44	46.65	14	9
FA0CS60	10.00	2.35	5	17.44	43.48	9	7
FA0CS80	11.99	2.25	6	28.96	14.83	14	8
FA0CS100	16.00	4.09	8	24.98	40.28	12	11
FA10CS0	16.02	14.65	8	33.91	69.90	16	7
FA10CS20	16.00	3.77	8	28.85	23.57	14	8
FA10CS40	20.0	4.0	10	34.14	23.99	17	8
FA10CS60	20.0	5.17	10	30.24	52.27	15	8
FA10CS80	17.99	3.88	9	33.80	41.21	17	12
FA10CS100	11.98	2.32	6	27.09	51.50	14	11
FA20CS0	10.04	3.37	5	25.71	35.42	13	14
FA20CS20	14.00	3.51	7	28.17	76.38	14	9
FA20CS40	14.00	2.92	7	28.34	72.78	14	7
FA20CS60	18.00	3.86	9	32.46	41.02	16	8
FA20CS80	16.00	3.72	8	35.4	60.00	18	10
FA20CS100	16.01	3.3	8	32.45	58.33	16	9
FA30CS0	6.00	1.09	3	27.08	52.04	14	7
FA30CS20	6.00	1.16	3	28.87	63.47	14	8
FA30CS40	13.99	2.25	7	33.18	51.42	17	6
FA30CS60	11.99	2.51	6	33.53	54.78	17	8
FA30CS80	11.94	2.51	6	34.98	55.24	17	10
FA30CS100	10.00	2.33	5	26.70	59.05	13	10

## Data Availability

Data sharing is not applicable.
